# Hereditary hypofibrinogenemia: A rare cause of chronic liver disease

**DOI:** 10.1002/jpr3.70058

**Published:** 2025-06-27

**Authors:** Hannah Caringal, Nolan Maloney, Khyati Mehta, Akshat Jain, Kalyan Parashette

**Affiliations:** ^1^ Loma Linda University Children's Hospital Loma Linda California USA

**Keywords:** coagulopathy, fibrinogen, RiaSTAP

## Abstract

Hypofibrinogenemia is characterized by low levels of fibrinogen with patients commonly presenting asymptomatically. This report discusses a case of hereditary hypofibrinogenemia manifesting as chronic liver disease in a 2‐year‐old male who was evaluated for elevated liver enzymes and skin/soft tissue bleeding.

## 1 INTRODUCTION

Hereditary hypofibrinogenemia involves dysfunctional protein aggregation in the endoplasmic reticulum, causing reduced levels of circulating fibrinogen. This mutant fibrinogen may cause bleeding or liver dysfunction, but typically, this disorder is discovered incidentally via lab results as patients are asymptomatic. Here, we present a case with elevated liver enzymes.

## 2 CASE REPORT

A 2‐year‐old male born prematurely at 32 weeks with gastric organo‐axial volvulus status post gastropexy and gastrostomy tube (GT) placement was evaluated in clinic for elevated transaminases since infancy. His family history was significant for paternal Marfan syndrome and maternal congenital hypofibrinogenemia confirmed by finding a pathogenic variant in the FGG gene (c. 1083G>T). His medical history was significant for multiple hospital admissions for slow feeding, coagulopathy, and anemia. On the physical exam, he appeared small for his age with a nondistended abdomen and a low‐profile GT in place. He had no bruising or hemorrhagic manifestations on his skin.

Lab work‐up demonstrated aspartate aminotransferase (AST) and alanine aminotransferase (ALT) ranging from 150 to 200 U/L (AST range: 39–228, normal 0–35; ALT range: 35–366; normal 0–35), normal total bilirubin, low fibrinogen levels in the 50 s mg/dL (normal 200–393), international normalized ratio (INR) of 1.2 (range 1.1–2.5; normal 0.8–1.1), and a lactate to pyruvate ratio of 33 (normal 10–20). Abdominal ultrasound revealed hepatomegaly with a liver span of 12.2 cm and normal echogenicity. Extensive work‐up including newborn screening, serology for Hepatitis A–C, Epstein‐Barr virus polymerase chain reaction (PCR), respiratory viral panel, COVID PCR, Immunoglobulin G level, ferritin level, and thyroid‐stimulating hormone levels were unremarkable. Antinuclear antibody, smooth muscle antibody, and anti‐liver‐kidney‐microsomal antibody were negative. Serum amino acid profile, total and free carnitine levels, acylcarnitine profile, and a 112‐gene cholestasis panel were normal. Alpha‐1 antitrypsin phenotype was the most common genetic combination for alpha‐1 antitrypsin alleles.

**Figure 1:**
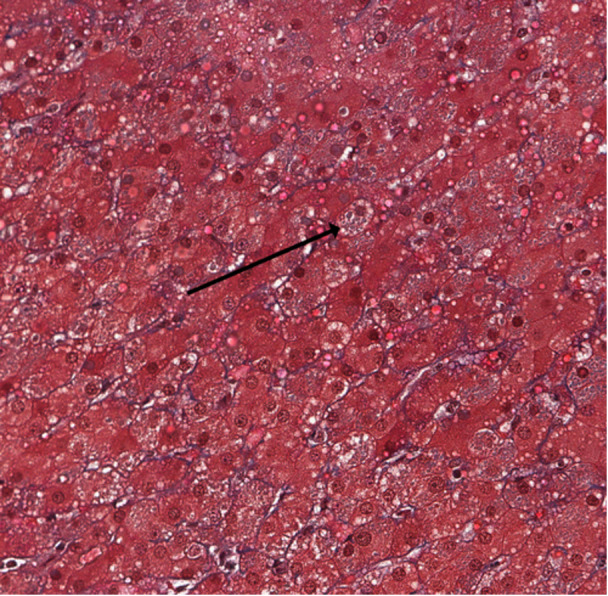
Trichome stain demonstrating multiple electron‐dense inclusions within the hepatocyte cytoplasm.

**Figure 2:**
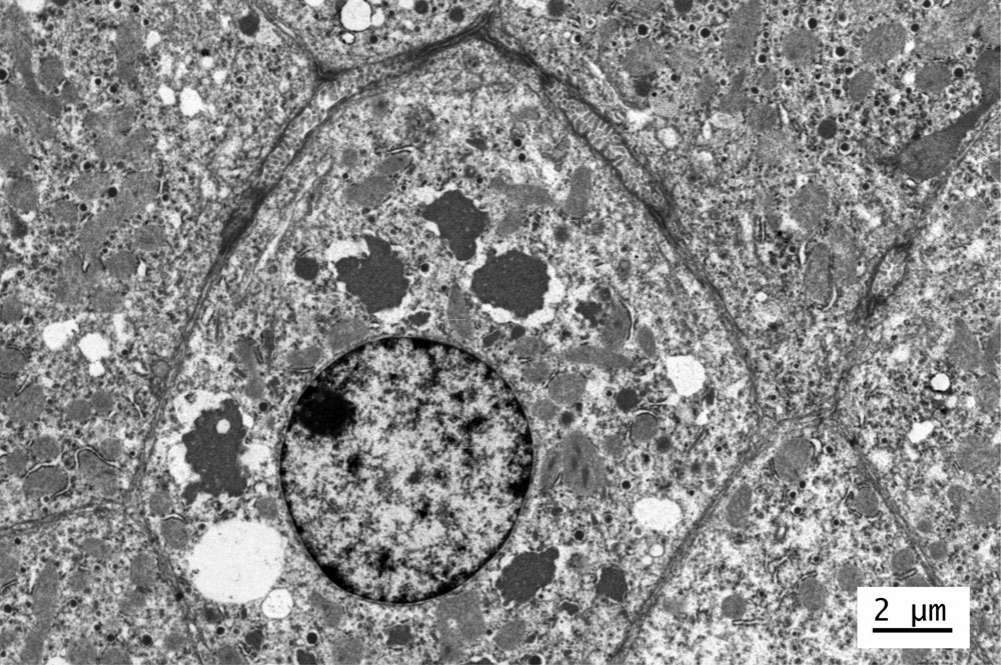
Electron microscopy showing electron‐dense inclusions within the hepatocyte cytoplasm.

**Figure 3:**
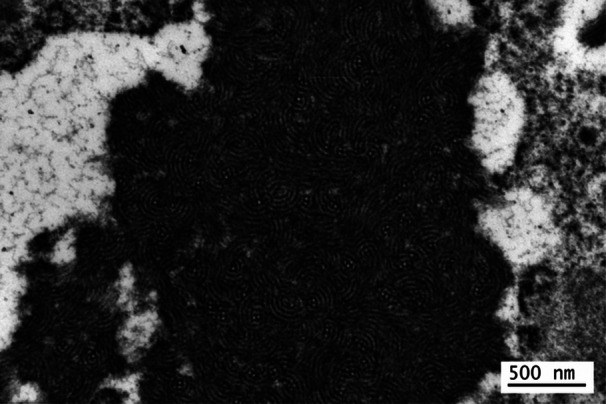
High power electron microscopic image showing electron‐dense inclusions possessing a fingerprint‐like morphology consistent with fibrinogen.

Liver biopsy demonstrated a trabecular arrangement of hepatocytes with a preserved lobular architecture and no significant inflammation, fibrosis, or steatosis. The trichrome histochemical stain revealed multiple electron‐dense inclusions within the hepatocyte cytoplasm (Figure 1). The electron microscopic exam demonstrated these inclusions to be composed of a particularly organized material with a fingerprint‐like morphology (Figures 2 and 3). This ultrastructural morphology is characteristic of fibrinogen.^1^ As hepatocytes work to produce and release rather than store fibrinogen, the presence of visible intracellular fibrinogen was consistent with genetic testing for hereditary hypofibrinogenemia with hepatic storage (HHHS).

**Figure 4:**
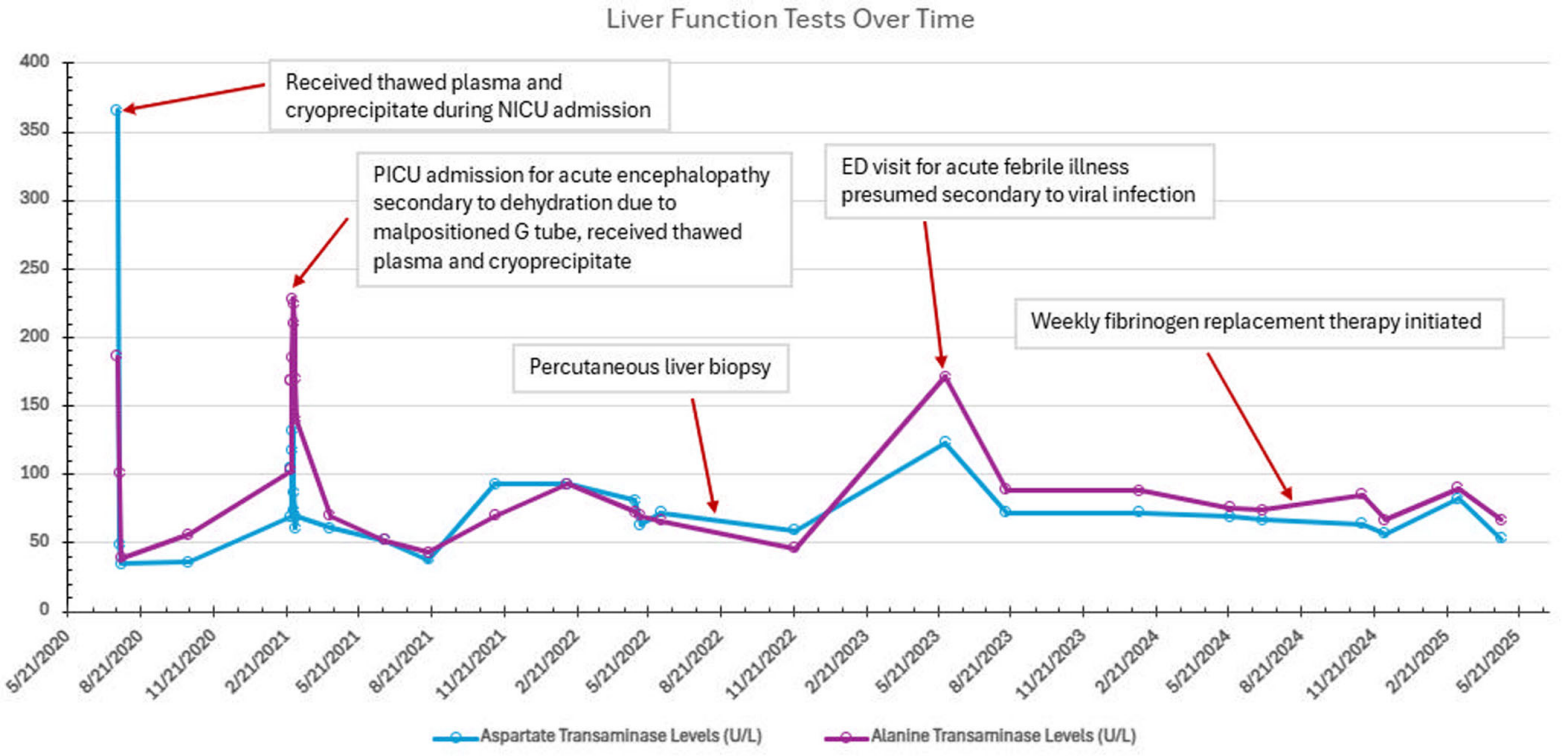
A timeline demonstrating liver function trends over the course of this patient's care.

Upon initiation of erythromycin and a feeding regimen via GT that allowed the patient to grow, his liver transaminases gradually stabilized in the 60–70 s U/L (Figure 4). He also began a regimen of weekly intravenous transfusions of Ria STAP (recombinant fibrinogen concentrate) to increase his fibrinogen level with a goal of >150 mg/dL. As this has improved his bleeding response, discussions for port placement have begun.

## 3 DISCUSSION

Fibrinogen is a glycoprotein encoded by three genes (fibrinogen alpha chain, fibrinogen beta chain, and fibrinogen gamma chain) that has an integral role in hemostasis, especially for blood clotting, wound healing, platelet adhesion, and aggregation.^2^ Most cases of hereditary hypofibrinogenemia result from heterozygous missense mutations of FGG, a gene associated with hepatic storage. The liver produces and secretes fibrinogen. While patients with hereditary hypofibrinogenemia are typically asymptomatic, some present with hematologic issues such as thromboembolism and, rarely, liver disease. In HHHS, mutated fibrinogen aggregate in hepatic cells, disrupting typical secretion and function.

In one study where 250 cases of dysfibrinogenemia gathered, 55% were asymptomatic.^3^ As there are thought to be over 500 cases reported since 1958, the incidence and prevalence of this disorder has not been well‐studied, especially the rarer incident of chronic liver disease.^4^ Of the few reported cases, HHHS appears to affect males and females equally and is usually discovered incidentally via mild to moderately elevated liver enzymes.^2^ HHHS is not widely prevalent but can present on a broad spectrum of liver disease, from normal hepatic function to severe chronic liver disease with fibrosis or cirrhosis. Typically, coagulation lab results may be abnormal, but hemorrhage or abnormal wound healing is not observed. The clinical presentation of this patient with skin and soft tissue bleeding in addition to increased AST/ALT levels demonstrates yet another variation of how this condition may manifest.

There is no protocol for management of HHHS; however, there are some reports that carbamazepine and ursodeoxycholic acid may decrease transaminase levels. One hypothesis poses that fibrinogen aggregating in the endoplasmic reticulum may lead to impaired autophagy and, therefore, medications that enhance autophagy (such as carbamazepine) can improve hepatic function.^5^ Long‐term management requires consistent monitoring of fibrinogen levels to prevent bleeding complications. Bleeds are controlled either with tranexamic acid or fibrinogen concentrate (cryoprecipitate).

## 4 CONCLUSION

Although HHHS does not occur commonly, this diagnosis should not be overlooked when evaluating a pediatric patient with elevated liver enzymes, particularly if the clinical picture is further clouded by vague manifestations of hematologic and hepatic dysfunction. Thorough investigation including lab workup, genetic testing, and, if appropriate, liver biopsy is warranted. The earlier this diagnosis is confirmed, the sooner patients can be more effectively treated with a multidisciplinary team of hematologists and hepatologists, leading to improved quality of life. Further research is also necessary to continue improving and standardizing care for pediatric patients with HHHS.

## CONFLICT OF INTEREST STATEMENT

The authors declare no conflict of interest.

## ETHICS STATEMENT

Informed patient consent was obtained from parent/guardian for publication of the case.
